# President's Address

**Published:** 1907-07

**Authors:** Geo. A. Esterly

**Affiliations:** Kansas State Dental Association.


### PRESIDENT'S ADDRESS.

By Geo. A. Esterly, Kansas State Dental Association.

Ethics, the meaning condensed, is the art of directing the actions of individuals for the benefit of society. Article II,

Section III, of our code, is referred to more than any other, and reads, "It is unprofessional to resort to public advertisements, cards, hand-bills, posters or signs calling attention to peculiar styles of work, lowness of prices, special modes of operating or to claim superiority over neighboring practitioners, to publish reports of cases or certificates in the public prints, to go from house to house to solicit or perform operations, to circulate or recommend nostrums or perform any similar acts."

This article has easy limitations and if a person wants to come just within its prescribed sphere and complies with the requirement of this code just to keep out of the throes of an offender, he is not an ethical man. Laws do not make ethical men. It is the high moral and conscientious man who does right because it is right and does it from the bottom of his heart. That is the truly ethical man.

A judge in our court, not long since, told one of our foremost citizens in a case that came before him, "You are technically within the law and right, but morally you are a thief." There is no question but that many of us are offenders in the same way. Technically right, but morally wrong.

Nothing of value has ever come to us from ancient workers in dentistry. Our profession has made more rapid progress than any other in the same length of time. Just think of it. We can almost claim the distinction of a complete evolution in a human life-time. To whom do we owe all of this? To the unselfish ethical man, whose highest ambition is to elevate his profession; who had no secrets, and bequeathed all he knew to us. All we have was his. Why will we not follow his example? Our profession today would be in the dark ages if those early practitioners, whose secrets were their fortunes, had the throttle in their hands for the future destiny of dentistry. We stand today on this tripod; the colleges, the journals and the societies. So it is to our best interest to do all that we can to promote their standard.—

—Western Dental Journal.

